# Trochlear Replacement Alone Without Patella Resurfacing in Patellofemoral Osteoarthritis: A Single Tertiary Centre Clinical Series

**DOI:** 10.7759/cureus.98073

**Published:** 2025-11-29

**Authors:** Meraj Akhtar, Uday Mahajan, Shahbaz S Malik, Tarek Boutefnouchet, Tanweer Ashraf

**Affiliations:** 1 Trauma and Orthopaedics, Nottingham University Hospitals NHS Trust, Nottingham, GBR; 2 Trauma and Orthopaedics, University Hospitals Birmingham NHS Foundation Trust, Birmingham, GBR; 3 Trauma and Orthopaedics, Worcestershire Acute Hospitals NHS Trust, Worcester, GBR; 4 Trauma and Orthopaedics, Royal Orthopaedic Hospital and University Hospitals Birmingham NHS Foundation Trust, Birmingham, GBR

**Keywords:** koos: knee injury and osteoarthritis outcome score, oxford knee scoring (oks), patellofemoral arthroplasty, patellofemoral joint osteoarthritis, patient outcomes, trochlear resurfacing

## Abstract

Background

Patellofemoral arthroplasty (PFA) is an established treatment for isolated patellofemoral joint osteoarthritis (PFJ OA). The procedure involves resurfacing the degenerated trochlear as well as the articular surface of the patella. In some patients with severe patellofemoral joint arthritis, the patella bone stock can be too thin to hold a plastic button. The patella in these cases could have a high fracture risk if resurfaced. We present the clinical outcome of a series of patients with severe patellofemoral joint arthritis where the trochlear alone was resurfaced, and the patella was too thin to be resurfaced.

Methods

All patients between January 2019 and December 2024 undergoing patellofemoral joint replacement in our unit were reviewed. All the patients who had trochlear replacement alone (without resurfacing of the patella) with a minimum follow-up of one year were included in this study. Six patients met the inclusion criteria. Functional outcomes were assessed using the Oxford Knee Score (OKS) and Knee Injury and Osteoarthritis Outcome Score (KOOS). Implant survivorship was determined through hospital records and cross-referenced with the National Joint Registry (NJR).

Results

The total number of patellofemoral joint replacements in our unit undertaken by a single surgeon (TA) was 51 from January 2019 to December 2024. Patella was not resurfaced (isolated trochlear replacement) in seven cases; six were included in our study, as one was lost to follow-up. The mean age at surgery was 54.7 years, and the mean follow-up was 39 months. All patients received Zimmer Gender Solutions (Zimmer Inc., Warsaw, IN) prosthesis. No patient required revision surgery until the last follow-up. Mean OKS at the last review was 34.16 (range 15-41), and mean KOOS was 39.78 (range 9.19-69.9). All patients experienced clinically significant improvement. No complications, reoperations, or radiographic progression of tibiofemoral osteoarthritis were observed.

Conclusion

Isolated trochlear replacement for severe patellofemoral arthritis without patella resurfacing is a safe option with reasonable short-midterm results. Larger prospective studies are needed to confirm long-term results.

## Introduction

Isolated patellofemoral joint osteoarthritis (PFJ OA) accounts for a significant proportion of symptomatic knee arthritis and remains a challenging condition to treat. Non-operative management, including physiotherapy, activity modification, and intra-articular injections, is often the first-line approach, but many patients experience persistent pain and functional limitation. Total knee arthroplasty (TKA) as a treatment option in isolated PFJ OA has been criticised as overtreatment because it sacrifices normal tibiofemoral compartments and cruciate ligaments. Moreover, 6-17% of patients experience persistent anterior knee pain after TKA for isolated patellofemoral arthritis [1,2]. Patellofemoral arthroplasty (PFA) offers several potential advantages compared to TKA, including preservation of bone stock and native tibiofemoral kinematics, shorter operative times, and faster recovery [3-6]. It is of particular interest in young, active patients because of easy conversion to TKA if necessary [3,7], resulting in better postoperative patient-reported outcome measures (PROMS) and range of motion (ROM) comparable to primary TKA [8].

A key technical controversy in PFA is the management of the patella when it is too thin to resurface [9]. Resurfacing can address advanced patellar cartilage loss but carries risks of fracture, maltracking, loosening, and overstuffing of the patellofemoral joint. Avoiding patella resurfacing avoids these complications, but concerns persist regarding residual anterior knee pain and lack of range of movement. Modern prostheses such as the Avon (Stryker, Howmedica Osteonics, Allendale, NJ) and Zimmer Gender Solutions (Zimmer Inc., Warsaw, IN) have demonstrated favourable outcomes [8].

Literature on the outcomes of PFA without patella resurfacing remains limited [9,10], and much of the available evidence comes from larger mixed series where patellar management is not clearly stratified and was left intact. We present a case series from a tertiary referral centre describing the outcomes of isolated trochlear replacement where the patella was too thin to be resurfaced with isolated PFJ OA. This study aims to add to the evidence base regarding the safety and effectiveness of leaving the patella un-resurfaced in the context of modern trochlear replacement.

## Materials and methods

This retrospective case series was conducted at the Royal Orthopaedic Hospital, Birmingham, United Kingdom, and included all patients who underwent isolated trochlear resurfacing for PFJ OA between January 2019 and December 2024. Institutional approval was obtained, and the study was performed in accordance with the Declaration of Helsinki. All patient data were collected from hospital electronic records and cross-referenced with the National Joint Registry (NJR) to ensure capture of any revision procedures outside the institution.

During the study period, a total of 51 PFAs were performed by a single senior arthroplasty surgeon with a specialist interest in patellofemoral surgery. Of these, seven patients underwent isolated trochlear replacement without patellar resurfacing. One patient was lost to follow-up, leaving six patients in the final analysis. The mean age at surgery was 54.7 years, ranging from 44.2 to 67 years. Five patients were female, and one was male. The right knee was operated on in two cases, and the left in four. All patients received the Zimmer Gender Solutions prosthesis.

Patients were included if they had radiographically and clinically confirmed isolated PFJ OA, were between 18 and 70 years of age, and had failed non-operative management such as physiotherapy, intra-articular injections, and oral analgesia. Patients were excluded if they had tibiofemoral osteoarthritis requiring TKA, previous patellectomy, inflammatory arthritis, active infection, or incomplete follow-up of at least 12 months. All operations were performed under tourniquet control using a standard medial parapatellar approach. A single dose of intravenous antibiotic was administered according to hospital protocol. A pre-contoured trochlear component was implanted in all cases using Palacos bone cement (Schering-Plough, Labo, Belgium). The patella was not resurfaced in any patient; if intraoperative calliper measurement demonstrated a patellar thickness of less than 20 mm, resurfacing was avoided to maintain a residual bone stock of at least 12 mm. Standard patelloplasty was performed in each case, consisting of removal of osteophytes and circumpatellar denervation. No patient required lateral release or facetectomy.

Postoperatively, patients followed a standardised rehabilitation program that allowed immediate mobilisation and weight bearing as tolerated. Follow-up assessments were conducted at six weeks, three months, one year, and annually thereafter. Standard anteroposterior, lateral, and skyline radiographs were obtained at each review to assess component alignment, loosening, and progression of tibiofemoral osteoarthritis.

The primary outcome was implant survivorship, defined as the absence of revision surgery, including conversion to TKA or secondary patellar resurfacing. Secondary outcomes included PROMS, specifically the OKS and the KOOS, as well as radiographic progression of tibiofemoral arthritis and any intraoperative or postoperative complications. Demographic variables such as age, sex, laterality, and body mass index (BMI) were also recorded.

As this study represents a retrospective review of all available cases meeting the inclusion criteria during the study period, no a priori sample size calculation was performed. The sample size reflects the rarity of the indication rather than a predefined statistical target. Formal power analysis was not feasible because of the absence of published data specifically addressing trochlear-only resurfacing without patellar replacement. However, prior systematic reviews of modern onlay PFA report revision rates between 4% and 10% at five years [3,11]. Based on these reported incidences, this cohort provides complete institutional coverage of all cases performed over the study period and serves as preliminary data for future power calculations. Statistical analysis was descriptive in nature; continuous variables were summarised using means and ranges, and categorical variables were expressed as frequencies and percentages. Given the small sample size, no hypothesis testing or regression modelling was performed.

## Results

During the six-year study period from January 2019 to December 2024, 51 patellofemoral arthroplasties were performed by the senior author. Seven of these were isolated trochlear replacements without patellar resurfacing, and one patient was lost to follow-up, leaving six patients for final analysis. The mean age at surgery was 54.7 years (range 44.2-67), and the cohort included five females and one male. Two right knees and four left knees were operated upon. All patients received the Zimmer Gender Solutions trochlear prosthesis.

All patients completed a minimum of 12 months of follow-up, with an average follow-up duration of 39 months. At the time of the last review, none of the patients had required revision surgery, and therefore revision-free survivorship was 100%. There were no intraoperative complications, wound problems, infections, thromboembolic events, or reoperations recorded during the follow-up period. Radiographic evaluation demonstrated stable components with no signs of loosening, lucency, or progression of tibiofemoral osteoarthritis in any case. No radiographs showed overstuffing or patellar maltracking.

Functional outcomes improved in all patients compared with their preoperative state. The mean OKS at final follow-up was 34.16 (range 15-41), and the mean KOOS was 39.78 (range 9.19-69.9). Two patients reported mild to moderate anterior knee pain at the final review, but all six described improved knee function and ability to perform daily activities compared to their preoperative condition. None of the patients required secondary patellar resurfacing or further surgical intervention.

A summary of patient demographics, implants, and clinical outcomes is presented in Table 1.

**Table 1:** Patient characteristics and outcomes

Variable	Value (n = 6)
Age (years), mean (range)	54.7 (44.2-67)
Sex, n	Female 5, male 1
Side operated, n	Right 2, left 4
Implant used	Zimmer Gender Solutions (all cases)
Follow-up duration (months), mean (range)	39 (12-72)
Revision-free survivorship	100%
Mean Oxford Knee Score (OKS)	34.16 (range 15-41)
Mean Knee Injury and Osteoarthritis Outcome Score (KOOS)	39.78 (range 9.19-69.9)
Residual anterior knee pain, n	2 mild to moderate
Radiographic progression of tibiofemoral OA	None
Complications or reoperations	None

Overall, the results demonstrate satisfactory short-term to midterm outcomes following isolated trochlear resurfacing without patellar replacement. All patients reported subjective improvement, and none required revision surgery during the study period. The absence of complications or radiographic deterioration supports the safety and feasibility of this approach in carefully selected patients with isolated PFJ OA.

## Discussion

The ideal procedure for isolated patellofemoral arthritis is controversial. PFA is an option that aims to restore the normal functions of the knee while preserving the bone. PFA has been shown to have benefits compared with TKA but has historically had a high failure rate. Revision rates are improving with modern implants and good indications, but remain higher than TKR [11].

PFA prostheses were first introduced in the 1970s, but their initial designs were associated with high failure rates, primarily due to the inlay design of the trochlear component [10,12]. This case series demonstrates that isolated trochlear resurfacing without patellar resurfacing can provide reliable survivorship and functional improvement at short-term to midterm follow-up. In this cohort of six patients, no revisions were recorded at a mean of just over 39 months, and all patients reported functional gains, although some degree of anterior knee pain persisted in two cases. Importantly, no radiographic progression of tibiofemoral osteoarthritis requiring further surgery was observed, and there were no intraoperative or postoperative complications.

Our findings are consistent with published evidence from modern PFA designs such as the Avon and Zimmer Gender Solutions [9,13]. In a recent systematic review pooling 21 case series and registry data, survivorship of PFA was reported as 90% at five years and 82% at 10 years, with progression of tibiofemoral arthritis being the commonest cause for revision rather than patellar management strategy [9,14]. There are some encouraging results from published series where the patella was not resurfaced as a standard practice [10,15].

Imhoff and colleagues [13,16] in a multicentre study of more than 260 patients highlight the interaction between implant design and patellar management. Our series adds to this body of evidence, supporting the practice of not resurfacing the patella in PFJ osteoarthritis in cases where the patella is too thin to do so.

Biomechanical studies provide further support for this distinction. Experimental work on limited trochlear resurfacing has shown that untreated defects increase patellofemoral contact forces and edge loading, while resurfacing can restore more physiological contact pressures [17-19]. Although the mean OKS and KOOS postoperative scores in our series were low, comparable to other PFJ series [11,16,17], and two patients had anterior knee pain, all patients reported functional benefit. None of the patients needed revision till the last follow-up.

The strengths of this series lie in its clear focus on unresurfaced patella, where it is too thin to do so in the setting of trochlear replacement, an area under-represented in the literature. The absence of revision procedures in our series is reassuring, and the improvement in patient-reported outcomes, even in a small cohort, is clinically relevant. Nevertheless, the limitations acknowledged are the retrospective design and small sample size, which limit generalisability. Longer-term follow-up and larger multicentre studies will be required to confirm whether these encouraging outcomes are sustained and whether certain patient or implant factors predict success when the patella is left unresurfaced.

## Conclusions

This case series demonstrates that isolated trochlear resurfacing without patella resurfacing can achieve satisfactory short to medium-term outcomes in patients with PFJ OA where the patella is too thin to resurface. Larger prospective studies are needed to define which patients benefit most from patella retention and to clarify the long-term durability of this approach.

## References

[1] Martinez-Lozano J, Martorell-de Fortuny L, Martin-Domínguez LA (2025). Patellofemoral arthroplasty provides similar long-term survival rate and complications with better clinical outcomes compared to facetectomy for the treatment of isolated patellofemoral osteoarthritis. J Exp Orthop.

[2] Koutserimpas C, Saffarini M, Bonnin M, Hirschmann MT, Lustig S (2025). Optimizing the patellofemoral compartment in total knee arthroplasty: is it time for dynamic assessment?. Knee Surg Sports Traumatol Arthrosc.

[3] Vella-Baldacchino M, Chughtai D, Kow J (2025). Outcomes of patellofemoral joint arthroplasty: a systematic review of revision timelines and complication rates. J Orthop Surg Res.

[4] Coden G, Schoeller L, Smith EL (2024). Robot-assisted patellofemoral arthroplasty. JBJS Essent Surg Tech.

[5] Bond EC, Stauffer TP, Hendren S, Amendola A (2023). Modern patellofemoral arthroplasty. JBJS Rev.

[6] Batailler C, Putzeys P, Lacaze F, Vincelot-Chainard C, Fontalis A, Servien E, Lustig S (2023). Patellofemoral arthroplasty is an efficient strategy for isolated patellofemoral osteoarthritis with or without robotic-assisted system. J Pers Med.

[7] Batailler C, Libert T, Oussedik S, Zaffagnini S, Lustig S (2024). Patello-femoral arthroplasty- indications and contraindications. J ISAKOS.

[8] Cardenas C, Wascher DC (2024). Outcomes of isolated patellofemoral arthroplasty. J ISAKOS.

[9] Imhoff AB, Bartsch E, Becher C (2022). The lack of retropatellar resurfacing at index surgery is significantly associated with failure in patients following patellofemoral inlay arthroplasty: a multi-center study of more than 260 patients. Knee Surg Sports Traumatol Arthrosc.

[10] Tarassoli P, Punwar S, Khan W, Johnstone D (2012). Patellofemoral arthroplasty: a systematic review of the literature. Open Orthop J.

[11] Roos EM, Roos HP, Lohmander LS (1998). Knee Injury and Osteoarthritis Outcome Score (KOOS)--development of a self-administered outcome measure. J Orthop Sports Phys Ther.

[12] Murray DW, Fitzpatrick R, Rogers K (2007). The use of the Oxford hip and knee scores. J Bone Joint Surg Br.

[13] Sava MP, Neopoulos G, Leica A, Hirschmann MT (2023). Patellofemoral arthroplasty with onlay prosthesis leads to higher rates of osteoarthritis progression than inlay design implants: a systematic review. Knee Surg Sports Traumatol Arthrosc.

[14] Vella-Baldacchino M, Webb J, Selvarajah B, Chatha S, Davies A, Cobb JP, Liddle AD (2023). Should we recommend patellofemoral arthroplasties to patients?. Bone Jt Open.

[15] Patel A, Haider Z, Anand A, Spicer D (2017). Early results of patellofemoral inlay resurfacing arthroplasty using the HemiCap Wave prosthesis. J Orthop Surg (Hong Kong).

[16] Calliess T, Ettinger M, Schado S, Becher C, Hurschler C, Ostermeier S (2016). Patella tracking and patella contact pressure in modular patellofemoral arthroplasty: a biomechanical in vitro analysis. Arch Orthop Trauma Surg.

[17] Sabatini L, Schirò M, Atzori F, Ferrero G, Massè A (2016). Patellofemoral joint arthroplasty: our experience in isolated patellofemoral and bicompartmental arthritic knees. Clin Med Insights Arthritis Musculoskelet Disord.

[18] Elbardesy H, McLeod A, Gul R, Harty J (2022). Midterm results of modern patellofemoral arthroplasty versus total knee arthroplasty for isolated patellofemoral arthritis: systematic review and meta-analysis of comparative studies. Arch Orthop Trauma Surg.

[19] van der List JP, Chawla H, Zuiderbaan HA, Pearle AD (2017). Survivorship and functional outcomes of patellofemoral arthroplasty: a systematic review. Knee Surg Sports Traumatol Arthrosc.

[20] Remy F (2019). Surgical technique in patellofemoral arthroplasty. Orthop Traumatol Surg Res.

[21] Provencher M, Ghodadra NS, Verma NN, Cole BJ, Zaire S, Shewman E, Bach BR Jr (2009). Patellofemoral kinematics after limited resurfacing of the trochlea. J Knee Surg.

